# The role of social support in the psychological illness of women*

**DOI:** 10.1590/1518-8345.2877.3157

**Published:** 2019-07-18

**Authors:** Loraine Vivian Gaino, Letícia Yamawaka de Almeida, Jaqueline Lemos de Oliveira, Andreia Fernanda Nievas, Denise Saint-Arnault, Jacqueline de Souza

**Affiliations:** 1 Fundação Hermínio Ometto, Araras, SP, Brasil; 2 Universidade de São Paulo, Escola de Enfermagem de Ribeirão Preto, Centro Colaborador da OPAS/OMS para o Desenvolvimento da Pesquisa em Enfermagem, Ribeirão Preto, SP, Brasil; 3 University of Michigan, School of Nursing, Ann Arbor, MI, EUA; 4 Bolsista da Coordenação de Aperfeiçoamento de Pessoal de Nível Superior (CAPES), Brasil

**Keywords:** Social Support, Primary Health Care, Health Promotion, Women, Mental Health, Mental Disorders, Apoio Social, Atenção Primária à Saúde, Promoção da Saúde, Mulheres, Saúde Mental, Transtornos Mentais, Apoyo Social, Atención Primaria de Salud, Promoción de la Salud, Mujeres, Salud Mental, Trastornos Mentales

## Abstract

**Objective:**

to analyze the relationship between perception of social support and emotional and physical symptoms associated with psychiatric conditions among women.

**Method:**

a cross-sectional, quantitative study was carried out with a randomized random sample of 141 women attended at a Family Health Unit of the city of Ribeirão Preto/SP. A sociodemographic questionnaire, the Social Support Questionnaire and the Self-Report Questionnaire were used.

**Results:**

there was no association between sociodemographic characteristics and mental disorder, but between aspects such as low income and schooling. The exercise of professions culturally considered as of low prestige gave rise to some reflections related to gender inequality. There was a significant difference in the satisfaction scores between the women who reported or not the symptoms of tiredness and sadness and the number of supporters among those who reported or not the symptom of fatigue. Spouses and children were the most mentioned supporters, and having mental disorder was significantly associated with having no friends in the support network.

**Conclusion:**

issues related to gender equity and satisfaction with social support are important aspects of care. For the promotion of mental health, efforts must be made to make women feel more connected and supported by the supporters available in their social environment.

## Abstract (pt)

**Objetivo:**

analisar a relação entre percepção de apoio social e sintomas emocionais e físicos associados a quadros psiquiátricos entre mulheres.

**Método:**

estudo transversal, quantitativo, realizado com uma amostra aleatória randomizada de 141 mulheres atendidas numa Unidade de Saúde da Família do munícipio de Ribeirão Preto/SP. Foram utilizados questionário sociodemográfico, Questionário de Suporte Social e *Self Report Questionaire*.

**Resultados:**

não houve associação entre as características sociodemográficas e o transtorno mental, mas entre aspectos como baixa renda e escolaridade. O exercício de profissões culturalmente consideradas de baixo prestígio suscitou algumas reflexões relacionadas à desigualdade de gênero. Houve diferença significativa nos escores de satisfação entre as mulheres que referiram ou não os sintomas de cansaço e tristeza e do número de apoiadores entre as que referiram ou não o sintoma de cansaço. Cônjuges e filhos foram os apoiadores mais mencionados e ter transtorno mental foi significativamente associado a não ter amigos na rede de apoio.

**Conclusão:**

questões relacionadas à equidade de gênero e à satisfação com o apoio social são aspectos importantes para a assistência. Para a promoção da saúde mental deve-se empreender esforços para que as mulheres se sintam mais conectadas e amparadas pelos apoiadores disponíveis em seu entorno social.

## Abstract (es)

**Objetivo:**

analizar la relación entre la percepción de apoyo social y síntomas emocionales y físicos asociados a cuadros psiquiátricos entre mujeres.

**Método:**

estudio transversal, cuantitativo, realizado con una muestra aleatorizada de 141 mujeres atendidas en una Unidad de Salud de la Familia del municipio de Ribeirão Preto/SP. Se utilizaron cuestionario sociodemográfico, Cuestionario de Apoyo Social y el *Self Report Questionaire*.

**Resultados:**

no hubo asociación entre las características sociodemográficas y el trastorno mental, pero entre aspectos como baja renta y escolaridad. El ejercicio de profesiones culturalmente consideradas de bajo prestigio suscitó algunas reflexiones relacionadas con la desigualdad de género. Se observó una diferencia significativa en los escasos de satisfacción entre las mujeres que mencionaron o no los síntomas de cansancio y tristeza y del número de apoyadores entre las que mencionaron o no el síntoma de cansancio. Los cónyuges e hijos fueron los apoyadores más mencionados y el trastorno mental se asoció significativamente a no tener amigos en la red de apoyo.

**Conclusión:**

cuestiones relacionadas con la equidad de género y la satisfacción con el apoyo social son aspectos importantes para la asistencia. Para la promoción de la salud mental se debe emprender esfuerzos para que las mujeres se sientan más conectadas y amparadas por los apoyadores disponibles en su entorno social.

## Introduction

Social support has been described as the available aid in the physical, psychological, material needs and the encouragement provided by individuals who make up the contact network, that is, family members, friends, neighbors, co-workers and others^(^ ^1^ ^–^ ^2^ ^)^. The social support network, in turn, consists of the set of persons or institutions that the individual realizes that he/she can trust or count for the provision of care, love and values^(^ ^3^ ^)^.

Studies have been developed on the effects of social support on people's health, associating it with different health outcomes^(^ ^1^ ^–^ ^2^ ^,^ ^4^ ^–^ ^5^ ^)^. Such a construct has been related to better abstinence rates and decreased use of drugs, as well as to the abandonment of behaviors harmful to health^(^ ^6^ ^)^.

Social support also influences how the individual evaluates and deals with stress, acting as a buffering of its negative consequences^(^ ^2^ ^)^.

The term “perception of social support” refers to whom the individual considers as a potential source of help for different needs. This perception is influenced both by the existence, in fact, of the sources of support, by the availability of the supporters, as well as by socioeconomic, psychological, cultural and professional aspects and age group, health conditions, and gender^(^ ^7^ ^)^.

Regarding gender, previous studies have pointed out that women are more likely to seek, receive, and benefit from social support^(^ ^8^ ^–^ ^9^ ^)^. However, they have also pointed out that the responsibilities culturally attributed to women, such as the care of children, of the sick ones, of the elderly, and household chores often create situations in which they need to resort greatly to their support networks^(^ ^8^ ^–^ ^9^ ^)^. Thus, both in the scope of research and care, the perception of social support can be considered an indicator of mental health, especially among women.

Studies indicate that better social support rates are inversely related to mental disorders^(^ ^8^ ^,^ ^10^ ^)^. That is, social support may act as a protective factor for mental health, mitigating the symptoms related to these disorders.

Research on social support and psychic symptoms in women has been developed with pregnant women, women who have recently given birth, or who have a specific health condition, such as cancer^(^ ^5^ ^,^ ^10^ ^–^ ^12^ ^)^. In view of the above, the following research question is proposed: Is there a relationship between social support and psychic illness among women in the general population? Therefore, the objective of the present study was to analyze the relationship between the perception of social support and the emotional and physical symptoms associated with psychiatric conditions among women of the general population.

## Method

This is a cross-sectional quantitative study carried out with women attended at a Family Health Unit in the city of Ribeirão Preto. The coverage region of this unit includes disadvantaged areas with respect to health and social outcomes due to factors such as poverty, low schooling, lack of sanitation, and high levels of violence. The service offers care in the areas of childcare, prenatal, gynecology, nursing and home care, family planning, and preventive actions in the community, with strong performance of community health workers.

The total number of women registered in this unit is 786, and 441 were in the age group between 18 and 65 years. The study participants were women of this age group, who were treated at this primary health care service. The only exclusion criterion adopted was having a clinical condition that impaired participation in the study (auditory, visual or speech impairment).

For the sample calculation, the formula for population with known size was used, proposed in literature^(^ ^13^ ^)^:

$$n=\frac{p(1-p)Z^{2}N}{\varepsilon^{2}(N-1)+Z^{2}\text{p(1}–\text{p)}}$$

**Figure 1:**
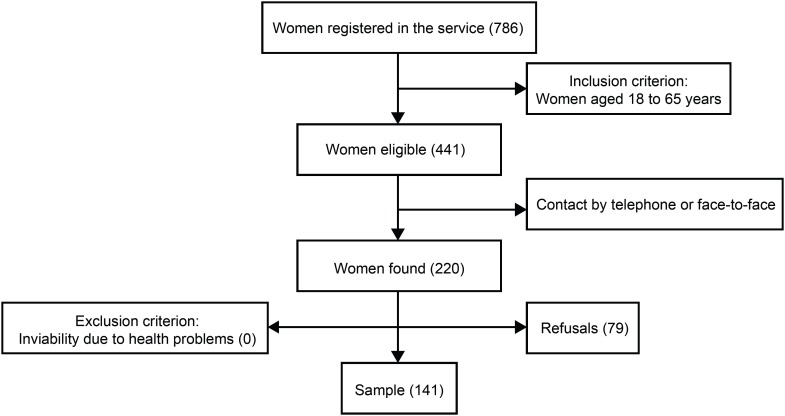
Diagram of constitution of the sample

The parameters used for the calculation were 95% confidence, 10% sample error and 44% estimated prevalence, considering a previous study on the prevalence of suspected cases of mental disorder among primary care women^(^ ^14^ ^)^. The estimated sample was 79 participants. A total of 220 women were invited and 141 accepted to participate, as shown in Figure 1. The main reason for refusal was the unavailability of time to respond to the questionnaire.

The randomization was done from a list with all the addresses of the families registered that had at least one woman. One woman from each randomly selected residence was invited face-to-face or by telephone to participate in the study. The data collection was carried out by a PhD student in psychology, a nurse with a master's level and two health workers from the same service who were trained for this data collection, which occurred during home visits or at the health unit, according to the preference of women.

The instruments of data collection were a sociodemographic questionnaire, the Social Support Questionnaire (SSQ) and the Self-Report Questionnaire (SRQ 20).

The SSQ, validated in Brazil, is composed of 27 questions and is divided into two parts. In the first one, the participant is asked to indicate the names of supporters for different situations, and can list from none to nine for each situation. Such information compose the social support score related to the number of supporters (SSQ-N). In the second part, the respondent reports his/her satisfaction with the support received, using a Likert scale, ranging from “very satisfied” (6) to “very dissatisfied” (1), composing the satisfaction score with the social support (SSQ-S)^(^ ^15^ ^)^.

The SRQ 20 was developed by the World Health Organization to assess symptoms of mental disorders in developing countries. The instrument is derived from four others: General Health Questionnaire (GHQ-60), Present State Examination (PSE), Post-Graduate Institute Health Questionnaire N 2 (PGI) and Patient Symptom Self-Report (PASSRo)^(^ ^16^ ^–^ ^17^ ^)^.

The original version has 24 items, in which the first 20 items evaluate non-psychotic disorders and the other four items, psychotic disorders. In the Brazilian version, as the study in which it was adapted was carried out in a Primary Health Care context, only the first 20 items are used^(^ ^16^ ^–^ ^17^ ^)^. The questionnaire has 20 “yes” or “no” questions about emotional and physical symptoms associated with psychiatric conditions. The sum of positive responses composes the final score (Score SRQ). Mental disorder cases are suspected when there are eight or more positive responses^(^ ^16^ ^)^.

The analyzes were carried out by a statistician, using R program, version 3.3.0. In the exploratory analysis, Pearson's Chi-square test or Fisher's exact test was used, considering the sociodemographic variables, the composition of the support network and the suspected cases of mental disorder. In relation to the variables symptoms and social support scores, the Mann Whitney test was used. To analyze the correlation between social support scores and SRQ scores, we used the Pearson correlation test.

The results of the exploratory analysis were the guiding factors for the delineation of a regression model. Thus, in the logistic regression analysis, the suspected mental disorder (score equal to or above eight points) was considered a dependent variable, classified as yes or no. The independent variables were self-reported color (white or yellow/black or brown), having children (yes/no), income (up to two minimum wages/above two minimum wages), exercising paid activity (yes/no), number of supporters (up to six/seven or more), satisfaction with support (yes/no), and presence of supportive friends (yes/no). From the adjusted regression model, the odds ratio and the corresponding confidence interval were calculated. The level of significance used in the analyzes was 5% (α = 0.05).

In order to carry out the study, all the ethical aspects provided for in Resolution 466/2012 of the National Health Council were met (Protocol CAAE-51267015.0.0000.5393).

## Results

Concerning the sociodemographic profile, the mean age of the participants was 43.4 years (SD = 13.3, median = 43). Most of them were married, white, Catholic, had one or two children, had no paid work, declared a family income of two to five minimum wages shared with approximately three people with whom they lived.

Among the participants, 44.7% (n = 63) had paid work, 64% (n = 91) reported having a profession and only 9% (n = 8) of these women referred to professions of higher prestige such as administrator, accountant, commercial manager, engineer, business owner and teacher. Professions that require formal qualification, such as nursing technician, administrative assistant, sales promoter and invoice provider, were mentioned by 32% (n = 29) of women, while less prestigious occupations, such as maids, cleaning women, housekeepers, general service providers, and clerks, corresponded to 59% (n = 54) of the mentioned professions.

The suspected cases of mental disorder corresponded to 43.4% (n = 61) of the sample and the distribution of these women according to their sociodemographic characteristics are presented in Table 1. As can be observed, there was a significant association between being a mother and having mental disorders.

**Table 1:** Distribution of participants according to sociodemographic characteristics and suspected cases of mental disorder (n = 141), Ribeirão Preto, SP, Brazil, 2017

Sociodemographic characteristics		Suspected case of mental disorder		Total n(%)	p-value
		Yes n(%)	No n(%)		
Marital status					
	Out of stable union	18(12.8)	31(22)	49(34.8)	
	In stable union	39(27.6)	49(34.7)	88(62.4)	0.388*
	Non-informed	04(2.8)		04(2.8)	
				
Cor					
	White or yellow	37(26.2)	44(31.2)	81(57.4)	
	Black or brown	24(17.0)	36(25.5)	60(42.5)	0.501*
				
Religion					
	Catholic	28(19.8)	40(28.4)	68(48.2)	
	Non-Catholic	33(23.4)	40(28.4)	73(51.8)	0.629*
				
Schooling					
	Incomplete high school or lower	35(24.8)	37(26.2)	72(51.0)	
	Complete high school or higher	26(18.4)	43(30.5)	69(49.0)	0.190*
				
Having children					
	Yes	58(41.1)	55(39.0)	113(80.1)	
	No	3(2.1)	25(17.7)	28(19.8)	**≤0.001** †
				
Exercise of paid activity					
	Yes	29(20.6)	34(24.1)	63(44.7)	
	No	31(22.0)	45(31.9)	76(53.9)	0.534*
	Non-informed	02(1.4)		02(1.4)	
				
Income					
	Up to 2 minimum wages‡	32(22.7)	37(26.2)	69(48.9)	
	Above 2 minimum wages‡	27(19.1)	42(29.8)	69(48.9)	0.390*
	Non-informed	03(2.1)		03(2.1)	

* Pearson's Chi-square test;

† Fisher's exact test;

‡ Brazilian minimum salary for the year 2017, which corresponded to R$ 937.00

Most of the interviewees were satisfied or very satisfied with their support network (average satisfaction score = 5.46, median = 5.7, SD = 0.72) and had six to nine supporters (mean = 7.7; median = 7; SD = 3.67). The most mentioned supporters were the children, the spouse and the parents. Of the total, only seven participants mentioned health professionals as supporters.

Table 2 shows the supporters mentioned according to the positive or negative status for mental disorder.

**Table 2:** Distribution of participants according to the composition of the support network and suspected cases of mental disorder (n = 141), Ribeirão Preto, SP, Brazil, 2017

Composition of support network		Suspected case of mental disorder		Total n(%)	p-value
		Yes n(%)	No n(%)		
Spouse					
	Yes	43(30.5)	58(41.1)	101(71.6)	
	No	18(12.8)	22(15.6)	40(28.4)	0.793*
Child					
	Yes	51(36.2)	50(35.5)	101(71.7)	
	No	10(7.1)	30(21.3)	40(28.4)	**0.006** *
Siblings					
	Yes	40(28.4)	51(36.2)	91(64.6)	
	No	21(14.9)	29(20.6)	50(35.5)	0.823*
Parents					
	Yes	39(27.7)	54(38.3)	93(66.0)	
	No	22(15.6)	26(18.4)	48(34.0)	0.658*
Other family members					
	Yes	32(22.7)	54(38.3)	86(61.0)	
	No	29(20.6)	26(18.4)	55(39.0)	0.070*
Friends					
	Yes	22(15.6)	45(31.9)	67(47.5)	
	No	39(27.6)	35(24.8)	74(52.4)	**0.017** *
Religion					
	Yes	04(2.8)	05(3.5)	09(6.3)	
	No	57(40.4)	75(53.2)	132(93.6)	0.941†
Co-workers					
	Yes	04(2.8)	06(4.3)	10(7.1)	
	No	57(40.4)	74(52.5)	131(92.9)	0.829†
Neighbors					
	Yes	02(1.4)	02(1.4)	04(2.8)	
	No	59(41.8)	78(55.3)	137(97.1)	0.783†
Health professionals					
	Yes	05(3.5)	02(1.4)	07(4.9)	
	No	56(39.7)	78(55.3)	134(95.1)	0.123†

* Pearson's Chi-square test;

† Fisher's exact test

In the exploratory analysis, considering all participants, a significant association between having mental disorder and not referring friends as supporters was identified. In addition, most women who had mental disorders mentioned their children as supporters, suggesting a possible confounding factor in relation to the outcome presented in Table 1 (association between having mental disorder and being a mother).

Thus, a new test was undertaken that aimed to analyze the validity of such association, considering only the mothers. It was identified that of the 113 women who had children, 12 did not mention them as supporters (seven with suspected disorder and five without it). The association between having mental disorder and mentioning children as supporters was not significant in the subgroup of the mothers participants (p = 0763 - Exact Fischer's Test).

Table 3 presents the most cited symptoms according to the social support score. As can be observed, women more satisfied with social support reported less symptoms of tiredness, sadness and were less likely to present a picture suggestive of mental disorder. There was a significant difference in the number of supporters only in relation to the fatigue symptom.

**Table 3:** Relationship between social support and emotional and physical symptoms associated with psychiatric conditions (n = 141), Ribeirão Preto, SP, Brazil, 2017

Suspicion of common mental disorder and major symptoms		Mean of social support score rank				
		n(%)	Satisfaction with support	p-value	Number of supporters	p-value
Do you nervous, tense or worried?						
	Yes	100(70.9)	67.5		71.65	
	No	41(29.1)	79.4	0.114*	69.43	0.768*
Have you felt sad lately?						
	Yes	72(51.1)	56.4		76.27	
	No	69(48.9)	86.2	**≤0.001** *	65.50	0.115*
Do you get tired easily?						
	Yes	59(41.8)	59.7		60.66	
	No	82(58.1)	79.1	**0.005** *	78.44	**0.010** *
Suspected case of mental disorder						
	Yes	61(43.3)	55.6		68.40	
	No	80(56.7)	82.7	**≤0.001** *	72.98	0.507*

* Mann-Whitney Test

**Figure 2:**
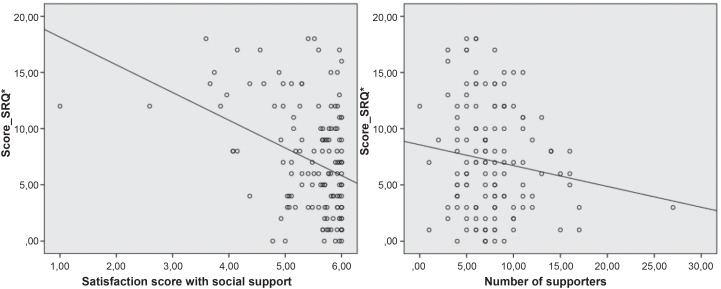
Correlation between score of satisfaction with social support, number of supporters and score of Common Mental Disorder * Score_SRQ – Self-Report Questionnaire Final Score

As can be observed in Figure 2, there was a negative correlation between satisfaction with support and suspicion of mental disorder (-0.374; p≤0.001). The correlation between this suspicion and the number of supporters was not significant (-0.142; p = 0.094).

The logistic regression analysis revealed that the satisfaction with the support and not having children were configured as protection factors in relation to the suspected mental disorder. That is, women who were not very satisfied with the received support [OR = 7.088 (CI 2.18-22.94), p = 0.001] and were mothers [OR = 7.2592 (CI 2.01-26.17), p = 0.002] had about seven times more chances of presenting a set of symptoms that characterized suspicion of mental disorder.

## Discussion

The results of the present study point to different issues related to gender inequality. One of them concerns the sociodemographic characterization of the participants, whose majority was married, with low family income and did not exercise paid activity. Those who had a paid activity generally referred to professions culturally considered of low prestige.

These results corroborate the discussion on gender and health, emphasizing that the opportunities, responsibilities and roles socially associated with being a man or woman are an axis of social differentiation that interacts with other attributes such as age, race, income, family structure, education, and social support^(^ ^18^ ^–^ ^20^ ^)^. Thus, there is the need to consider the intersection of these attributes in the research and planning of women's health actions, since it is an important determinant of health and mental health^(^ ^18^ ^–^ ^20^ ^)^.

The fact that the main symptoms mentioned by the participants are fatigue, sadness, and nervousness corroborates a previous study^(^ ^21^ ^)^. This result, analyzed in the light of sociodemographic characteristics, refers us to a situation of vulnerability that combines psychic and social aspects. The life context of these women contributes significantly to the increase of such symptoms, increasing the risks for presenting mental disorders.

Sadness, specifically, was mentioned by more than half of the participants of the present study. Although sadness alone does not determine a psychiatric condition, such symptom requires accurate contextualization and effective support for its management, since it contributes in an important way to the development of more severe conditions such as depression^(^ ^22^ ^)^.

Therefore, considering that women are at greater risk of developing depression^(^ ^23^ ^)^ and that emotional complaints tend to be neglected in the face of other health demands^(^ ^24^ ^)^, the development of qualified listening spaces in primary care is recommended so as to provide comprehensive and welcoming assistance to the population, especially for women.

In this sense, it is worth mentioning that health professionals were the least mentioned supporters. The Family Health Units differ from the traditional Basic Units by the organization of their work process, with emphasis mainly on the size of the coverage area, attention to clients’ specific characteristics, territorial approach, and work dynamics with periodic home visits^(^ ^25^–^26^ ^)^. In this logic, the proximity to the clientele and the closer ties with the community are crucial, assuming that such teams should be configured in effective references of support, especially in health-related needs^(^ ^25^–^26^ ^)^. Considering that all the participants were registered and have been accompanied by professionals of the Family Health Strategy and that the instrument used in the data collection mentioned some health-related issues, these professionals would be expected to be more relevant in the social support networks of these women.

The issue of access is thus an important element of this discussion, since the mere existence of a resource that can provide care or assistance does not necessarily guarantee that it is perceived as a supporter by the user^(^ ^27^ ^)^. Access to available resources implies aspects such as the type of reception offered, clarity about what the institution can provide the user, the resolution of the demands presented, and even the visibility of the role that the institution plays in the community^(^ ^27^ ^)^.

Regarding the suspected cases of mental disorder, the present study identified a lower percentage than the one indicated in previous studies carried out with women in the primary care level^(^ ^21^ ^,^ ^28^ ^)^. This difference may reflect the results related to social support. In this sense, the number and diversity of supporters, as well as the satisfaction with them, deserve to be highlighted because they were larger than those identified in previous research^(^ ^29^ ^)^.

Satisfaction with social support seems to play a protective role both in relation to the symptoms and the suspected disorder itself.

Friends and children were highlighted as supporters, corroborating studies that identified support from friends as a protective factor for women's mental health^(^ ^12^ ^,^ ^30^ ^–^ ^31^ ^)^. Thus, a network whose composition contains friends indicates a certain diversification in the sources of social support. Such a condition is considered beneficial to mental health, since friends can facilitate access to information and health services, encourage self-care, and provide more effective practical and/or emotional support in the face of family and/or marital problems^(^ ^32^ ^–^ ^35^ ^)^.

The results showed that having children constituted a significant risk factor in relation to the symptoms characteristic of mental disorders, corroborating previous research^(^ ^28^ ^,^ ^36^ ^–^ ^37^ ^)^. These results suggest that responsibilities with children, often attributed only to women, can contribute to task overload, high levels of stress, and increased symptoms of mental disorders^(^ ^36^ ^)^.

In spite of this, the children constituted one of the most mentioned groups of supporters, referring to the so-called “negative effect of social support”. This effect concerns a duplicity of role played by the supporter, that is, the individual or institution can either provide support or become a source of stress because of the ambiguous character that marks some interpersonal relationships^(^ ^36^ ^–^ ^39^ ^)^. Therefore, the negative effect of social support should also be taken into account in planning health actions and future research.

Corroborating previous studies^(^ ^11^ ^–^ ^12^ ^,^ ^40^ ^)^, the results also pointed out that social support is a protective factor of possible cases of mental disorders. On the other hand, unlike previous research on social support and mental disorder in women, the present study analyzed two aspects of this construct, namely the number of supporters and the satisfaction with the support received. Negative correlation was identified for both aspects, but statistical significance was only for satisfaction with the support. This result highlights the need to consider that quality is as important as the other characteristics of social support, especially in studies that have psychosomatic issues as their object.

In summary, the present research identified that the satisfaction with the social support has, in fact, a protective effect in relation to mental disorders. In addition, children are important supporters, although “being a mother” was a risk factor for mental disorder among the women studied.

Regarding the implications for the practice, there is a need to strengthen the bond between health professionals and users, since the establishment of links has been identified as promising for effective reception, improved communication among those involved, and facilitator in the identification of different health needs^(^ ^41^ ^–^ ^42^ ^)^. These aspects contribute to more comprehensive and supportive approaches and, consequently, to increase the perception of support by individuals.

In this sense, providing a listening that transcends the aspects traditionally considered clinical (those related to physical symptoms) and other actions that do not go beyond the scope of the skills of general practitioners could be adopted. Clarification on the care offered by the community service, especially those in the field of mental health, and the inclusion of emotional issues in the elaboration of individual Therapeutic Projects are some initiatives that, according to previous studies^(^ ^43^ ^–^ ^44^ ^)^, can contribute to broadening access to mental health care and to enabling health approaches from a more holistic perspective.

Strategies to improve the resolution of possible conflicts in the mother-child relationship are also important and could be achieved through qualified listening and support to the various maternal needs and distress in different life cycles. Qualified listening, as a light and relational technology, contributes to the individualization of the subjects and the extension of the technical capacity of the teams, especially in relation to the psychosocial demands in the community^(^ ^45^ ^–^ ^46^ ^)^. Another possibility would be the offering of rounds of conversation and group activities that provide the exchange of experiences between peers, as pointed out in previous studies^(^ ^47^ ^–^ ^49^ ^)^.

Promoting actions to improve stress management can also be relevant since it is associated with discussions on gender inequality in broader forums, aiming to dismantle the culture of assigning the role of caring for the family only to women, as well as combat the culture of submission, which permeates their daily lives. The importance of debating these themes is also reiterated in the literature^(^ ^50^ ^–^ ^51^ ^)^.

Thus, it is understood that such recommendations may be useful for primary care professionals and are within the scope of general practitioner skills. These actions may aid in broadening social support and reducing suffering that culminates in psychosomatic symptoms.

Among the limitations of this study, there is the fact that the sample was obtained only from one Family Health Unit, which makes it impossible to generalize the results. Although the gender cut was intentional in the present study, a survey that also included men could provide more conclusive results regarding the performance of the variable “having children” in the relation between satisfaction with support and possible case of mental disorder.

Despite these limitations, we believe that the aspects listed in this research are extremely important in guiding and assisting the consolidation of mental health promotion practices. In addition, they corroborate the agenda of priorities and recommendations of the national and international health agencies, especially in the sense of integrating the promotion of gender equity in the expansion of access to mental health actions in primary health care^(^ ^52^ ^–^ ^53^ ^)^.

Furthermore, with regard to the external validity of the results, given the profile of the participants and the context of gender and health inequality in most low-income and middle-income countries, it is understood that the discussion and recommendations raised in the present study are applicable also to women in situations of social vulnerability in other regions of Brazil and even in other developing countries.

## Conclusion

The development of this research identified that women who were less satisfied with social support were more susceptible to presenting psychiatric conditions. In addition, the results suggest that gender-based distribution of roles may be a contributing factor for women who have children to be more susceptible to mental disorders.

Thus, both issues related to gender equity and satisfaction with social support should be considered in the planning of actions aimed at the promotion of mental health, especially in primary health care.

The undertaking of efforts in this direction implies to promote care that contemplate the subjective scope of the users, so that they feel more connected and supported by the formal and informal supporters available in their social environment and in the territory in which they live.

## References

[1] Xu L, Song R (2016). Influence of work–family–school role conflicts and social support
on psychological wellbeing among registered nurses pursuing advanced
degree. Appl Nurs Res. [Internet].

[2] Stein ER, Smith BW (2015). Social support attenuates the harmful effects of stress in
healthy adult women. Soc Sci Med. [Internet].

[3] Perry BL, Pescosolido BA (2015). Social network activation: the role of health discussion partners
in recovery from mental illness. Soc Sci Med. [Internet].

[4] Silva SM, Braido NF, Ottaviani AC, Gesualdo GD, Zazzetta MS, Orlandi FS (2016). Social support of adults and elderly with chronic kidney disease
on dialysis. Rev. Latino-Am. Enfermagem. [Internet].

[5] Thompson T, Pérez M, Kreuter M, Margenthaler J, Colditz G, Jeffe DB (2017). Perceived social support in African American breast cancer
patients: Predictors and effects. Soc Sci Med. [Internet].

[6] Reed E, Emanuel AN, Myers B, Johnson K, Wechsberg WM (2013). The relevance of social contexts and social action in reducing
substance use and victimization among women participating in an HIV
prevention intervention in Cape Town, South Africa. Subst Abuse Rehabil. [Internet].

[7] Silva-Rocha VV, Oliveira CM, Shuhama R (2016). The perception of social support and depressive symptomatology in
young women assisted at a Family Health Center. Rev Bras Med Fam Comun. [Internet].

[8] Almeida LM, Costa-Santos C, Caldas JP, Dias S, Ayres-de-Campos D (2016). The impact of migration on women's mental health in the
postpartum period. Rev Saúde Pública. [Internet].

[9] Milner A, Krnjacki L, Lamontagne AD (2016). Age and gender differences in the influence of social support on
mental health: a longitudinal fixed-effects analysis using 13 annual waves
of the HILDA cohort. Public Health. [Internet].

[10] Corrigan CP, Kwasky AN, Groh CJ (2015). Social Support, Postpartum Depression, and Professional
Assistance: A Survey of Mothers in the Midwestern United
States. J Perinatol Educ. [Interent].

[11] Natamba BK, Mehta S, Achan J, Stoltzfus RJ, Griffiths JK, Young SL (2016). The association between food insecurity and depressive symptoms
severity among pregnant women differs by social support category: a
cross-sectional study. Matern Child Nutr. [Internet].

[12] Baumgartner JN, Parcesepe A, Mekuria YG, Abitew DB, Gebeyehu W, Okello F (2016). Correlates of postpartum common mental disorders: results from a
population-based study in Amhara region, Ethiopia. Arch Womens Ment Health. [Internet].

[13] Agranonik M, Hirakata VN (2011). Sample size calculation: proportions. Rev HCPA. [Internet].

[14] Borges TL, Miasso AI, Vedana KGG, Telles PCP, Hegadoren KM (2015). Prevalence in the use of psychotropics and associated factors in
primary health care. Acta Paul Enferm. [Internet].

[15] Matsukura TS, Marturano EM, Oishi J (2002). The Saranson's Social Support Questionnaire: studies regarding
its adaptation to portuguese. Rev. Latino-Am. Enfermagem. [Internet].

[16] Mari JJ, Willians P (1986). A validity study of a psychiatric screening questionnaire
(SRQ-20) in primary care in the city of São Paulo. Br J Psychiatry. [Internet].

[17] Santos KOB, Araújo TM, Oliveira NF (2009). Factor structure and internal consistency of the Self-Reporting
Questionnaire (SRQ-20) in an urban population. Cad Saúde Pública. [Internet]..

[18] Delara M (2016). Role of ethnography in exploring mental health experiences of
female muslim immigrant youths. J Ment Disord Treat. [Internet]..

[19] Oliveira HSB, Fumis RRL (2018). Sex and spouse conditions influence symptoms of anxiety,
depression, and posttraumatic stress disorder in both patients admitted to
intensive care units and their spouses. Rev Bras Ter Intensiva. [Internet]..

[20] Owusu M, Nursey-Bray M, Rudd D (2018). Gendered perception and vulnerability to climate change in urban
slum communities in Accra, Ghana. Reg Environ Change. [Internet]..

[21] Silva SSBE, Oliveira SFSB, Pierin AMG (2016). The control of hypertension in men and women: a comparative
analysis. Rev Esc Enferm USP. [Internet]..

[22] Gonçalves DA, Mari JJ, Bower P, Gask L, Dowrick C, Tófoli LF (2014). Brazilian multicentre study of common mental disorders in primary
care: rates and related social and demographic factors. Cad Saúde Pública. [Internet]..

[23] Velez DMA, Maquet YG, Lopez PL (2014). Psychometric Propierties of The State-Trait Depression Inventory
(IDER) with a Colombian general sample. Av Psicol. Latinoam. [Internet]..

[24] Fernandez A, Moreno-Peral P, Zabaleta-del-Olmo E, Bellon JA, Aranda-Regules JM, Luciano JV (2015). Is there a case for mental health promotion in the primary care
setting? A systematic review. Prev Med. [Internet]..

[25] Wenceslau LD, Ortega F (2015). Mental health within primary health care and Global Mental
Health: international perspectives and Brazilian context. Interface (Botucatu) [Internet]..

[26] Bezerra IC, Jorge MSB, Gondim APS, Lima LL, Vasconcelos MGF (2014). “I went to the health unit and the doctor sent me here”: process
of medicationalization and (non)resolution of mental healthcare within
primary care. Interface. (Botucatu) [Internet]..

[27] Souza J, Magalhães RC, Saint Arnault DM, Oliveira JL, Barbosa SP, Assad FB (2017). The Role of Social Support for Patients with Mental Disorders in
Primary Care in Brazil. Issues Ment Health Nurs. [Internet].

[28] Salinero-Fort M, Jiménez-García R, Burgos-Lunar C, Chico-Moraleja R, Gómez-Campelo P (2015). Common mental disorders in primary health care: differences
between Latin American-born and Spanish-born residents in Madrid,
Spain. Soc Psychiatry Psychiatr Epidemiol. [Internet].

[29] Goodman SH, Bakeman R, McCallum M, Rouse MH, Thompson SF (2017). Extending models of sensitive parenting of infants to women at
risk for perinatal depression. Parent Sci Pract. [Internet]..

[30] Santini ZI, Fiori KL, Feeney J, Tyrovolas S, Haro JM, Koyanagi A (2016). Social relationships, loneliness, and mental health among older
men and women in Ireland: A prospective community-based
study. J Affect Disord. [Internet]..

[31] Feng X, Astell-Burt T (2016). What types of social interactions reduce the risk of
psychological distress? Fixed effects longitudinal analysis of a cohort of
30,271 middle-to-older aged Australians. J Affect Disord. [Internet]..

[32] Souza J, Almeida LY, Moll MF, Silva LD, Ventura CAA (2016). Structure of the social support network of patients with severe
and persistent psychiatric disorders in follow-ups to primary health
care. Arch Psychiatr Nurs. [Internet]..

[33] Kyriakakis S (2014). Mexican immigrant women reaching out. Violence Against Women [Internet]..

[34] Molina Y, Ornelas IJ, Doty SL, Bishop S, Beresford SAA, Coronado GD (2015). Family/friend recommendations and mammography intentions: the
roles of perceived mammography norms and support. Health Educ Res. [Internet]..

[35] Hasson-Ohayon I, Goldzweig G, Sela-Oren T, Pizem N, Bar-Sela G, Wolf I (2015). Attachment style, social support and finding meaning among
spouses of colorectal cancer patients: Gender differences. Palliat Support Care. [Internet]..

[36] Marconato CS, Magnago ACS, Magnago TSBS, Dalmolin GL, Andolhe R, Tavares JP (2017). Prevalence and factors associated with minor psychiatric
disorders in hospital housekeeping workers. Rev Esc Enferm USP. [Internet]..

[37] Maier C, Laumer S, Eckhardt A, Weitzel T (2015). Giving too much social support: social overload on social
networking sites. Eur J Inf Syst. [Internet]..

[38] Song A, Wenzel SL (2015). The association of social networks with substance use among
homeless men in Los Angeles who have unprotected sex with
women. J Subst Use. [Internet]..

[39] Freisthler B, Holmes MR, Wolf JP (2014). The dark side of social support: Understanding the role of social
support, drinking behaviors and alcohol outlets for child physical
abuse. Child Abuse Neg. [Internet].

[40] Santos LM, Amorim LDAF, Santos DN, Barreto ML (2015). Measuring the level of social support using latent class
analysis. Soc Sci Res. [Internet]..

[41] Santos RCA (2016). Importance of the bond between profesional and user in family
health strategy. Rev Enferm UFSM. [Internet]..

[42] Lima EFA, Sousa AI, Cândida C Primo, Leite FMC, Lima RCD, Maciel ELN (2015). An assessment of primary care atributes from the perspective of
female healthcare users. Rev. Latino-Am. Enfermagem. [Internet]..

[43] Silva GR, Reis HFT, Santos EM, Souza MPA, Azevedo RL (2016). Mental health in primary care: Perceptions of the family health
care team. Cogitare Enferm. [Internet]..

[44] Gryschek GP, Pinto AAM (2015). Mental health care: how can Family Health teams integrate it into
Primary Healthcare?. Ciênc Saúde Coletiva. [Internet]..

[45] Torres GMC, Figueiredo IDT, Cândido JAB, Pinto AGA, Morais APP, Araújo MFM (2017). Therapeutic communication in the interaction between health
workers and hypertensive patients in the family health
strategy. Rev Gaúcha Enferm. [Internet]. [Internet]..

[46] Rocha MGL, Linard AG, Santos LVF, Sousa LB (2018). Embracement in gynecological nursing consultation: women's
perceptions of the Family Health Strategy. Rev Rene. [Internet]..

[47] Vasconcelos MIO, Farias QLT, Nascimento FG, Cavalcante ASP, Mira QLM, Queiroz MVO (2017). Health education in primary care: an analysis of actions with
hypertension patients. Rev APS. [Internet]..

[48] Silva MAM, Marques FM, Brito MCC, Viana RS, Mesquita ALM, Silva ASR (2018). Operative group of primigravidae: a health promotion
strategy. Rev Bras Promoção Saúde. [Internet].

[49] Ribeiro KG, Andrade LOM, Aguiar JB, Moreira AEMM, Frota AC (2018). Education and health in a region under social vulnerability
situation: breakthroughs and challenges for public policies. Interface. (Botucatu) [Internet].

[50] World Health Organization (2000). Women's Mental Health: an evidence based review. [Internet].Mental
Health Determinants and Populations. Department of Mental Helath and
Substance Dependence.

[51] Oliveira HSB, Fumis RRL (2018). Sex and spouse conditions influence symptoms of anxiety,
depression, and posttraumatic stress disorder in both patients admitted to
intensive care units and their spouses. Rev Bras Ter Intensiva. [Internet]..

[52] Abdulmalik J, Thornicroft G (2016). Community mental health: a brief, global
perspective. Neurol Psychiatry Brain Res. [Internet]..

[53] World Health Organization (2017). Mental Health Atlas. Mental Health Action Plan 2013-2020
[Internet].

